# TriENA: a Portable, Hybrid Multimode Spectrometer Combining Diffuse Reflectance, LED-Induced Fluorescence, and Laser-Induced Breakdown Spectroscopy for a Holistic Analysis of Materials on Monuments and Objects of Archaeological Interest

**DOI:** 10.1021/acs.analchem.5c05236

**Published:** 2025-12-05

**Authors:** Victor Piñon, Anastasia Giakoumaki, Michalis Andrianakis, Kostas Hatzigiannakis, Kristalia Melessanaki, Panagiotis Siozos, Demetrios Anglos, Paraskevi Pouli

**Affiliations:** † Institute of Electronic Structure and Laser, 124215 Foundation for Research and Technology - Hellas, Nikolaou Plastira 100, Vassilika Vouton, Heraklion, Crete GR-700 13, Greece; ‡ Department of Chemistry, University of Crete, Heraklion, Crete GR-710 03, Greece

## Abstract

In the context of modern Heritage Science (HS) studies, chemical analysis together with contextual information provide valuable insights into the composition and structure, provenance, age, and/or production technology of objects and materials, facilitating also decision-making concerning the selection of appropriate conservation approaches for artifacts and monuments. While powerful laboratory techniques are often employed in such investigations, an increasing demand exists for the use of portable instrumentation that can be deployed at archaeological sites or museums. To address these needs, TriENA, a portable, hybrid instrument, has been developed, which integrates three complementary spectrochemical tools: Laser-Induced Breakdown Spectroscopy (LIBS), LED-Induced Fluorescence (LED-IF), and Diffuse Reflectance (DR) spectroscopy. These techniques facilitate compositional analysis of different types of materials, including metals, alloys and their corrosion products, pottery, pigments in various types of painted objects, or the identification of biodeterioration on stone monuments. In this article, the design concept of TriENA is presented. Detailed technical information concerning the main components of the instrument is provided, and key engineering aspects are discussed. A series of test cases demonstrate that this hybrid spectrometer is a valuable and versatile tool for conservators, heritage scientists, and archaeologists, as it enables rapid, portable, and comprehensive characterization of a wide range of objects, addressing a broad spectrum of analytical challenges.

## Graphical abstract

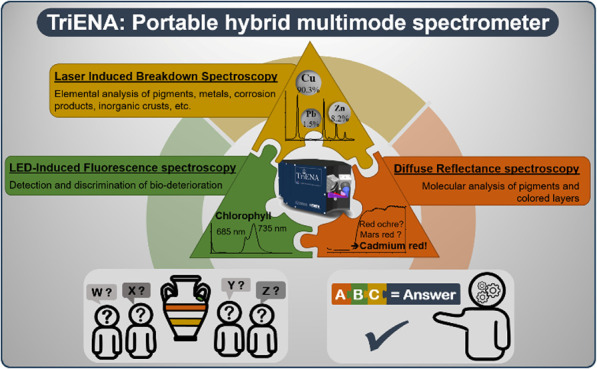

## Introduction

Heritage Science (HS) relies fundamentally on and benefits considerably from the use of a broad range of diagnostic and analytical tools. These are employed by researchers and practitioners, who want to gain information on the chemical composition and identity of various materials in different types of movable and immovable heritage objects, archaeological and historical findings, monuments, and building structures. These materials, clearly of very diverse composition, structure, and origin, may represent the main (bulk) constituents of the objects under investigation, for example, stone, glass, metal, paper, leather, wood, pigments, as well as human, animal, or plant remains. They might as well represent deposits of environmental or burial origin, products of recent or long-term degradation or corrosion processes, other types of remains or contaminants, and even traces related to object usage. As noted, the context and orientation of a given investigation may vary, and this dictates, by and large, specific questions, which are asked and consequently those analytical methods that may be more suitable to address such questions. For example, studies may have as their goals the elucidation of materials provenance or object dating, the determination of manufacturing techniques in relation to physical and chemical processing, the detection of forgeries, or the identification of archaeological residues, for instance, food products, cosmetics, or drugs.

In all, carrying out a well-planned analysis, targeted toward the identification and quantification of key components, is crucial for addressing effectively each specific problem. It is certainly understood that analytical information obtained by a given method illuminates only certain aspects of the overall material composition profile; hence, different analytical techniques used may be considered as providing a complementary piece of a puzzle. Together with other relevant pieces of information, such as, for example, knowledge of raw materials employed during a certain historical period or at a certain site, manufacturing technology, or object usage, results from chemical analysis facilitate our understanding and contextualization of objects and monuments that hold significant historical, artistic, and cultural value. In addition, compositional analysis may also provide valuable input to conservators, aiding them in making informed decisions in choosing appropriate restoration actions.

The key role of chemical analysis and associated methods and techniques in the context of heritage studies has been presented in multiple literature reviews and dedicated books.^1–6^ For obvious reasons, noninvasive or minimally invasive techniques are preferred when analyzing valuable heritage objects and, in this context, spectrochemical analysis methods are being increasingly employed in studies of heritage objects and monuments. These include Raman and FT-IR spectroscopy,^7–10^ X-ray fluorescence (XRF) spectroscopy,^11,12^ laser ablation inductively coupled plasma–mass spectrometry (LA-ICP-MS),^13,14^ Laser-Induced Breakdown Spectroscopy (LIBS),^15–17^ conventional and Laser-Induced Fluorescence (LIF) spectroscopy,^18,19^ as well as absorbance/reflectance spectroscopy^20^ or nuclear magnetic resonance (NMR) spectroscopy.^21^ Powerful analytical approaches make use of synchrotron and ion-beam facilities.^22–24^ Recently, important advances have been achieved by the use of high-resolution mass spectrometric methods, concerning the analysis of bio-organic proteinaceous materials^25,26^ while stable-isotope analyses have been increasingly employed in palaeodiet and population studies.^27^

The importance of highly sensitive and selective laboratory techniques, such as the ones highlighted above, is broadly recognized, and the use of such state-of-the-art instrumentation to approach complicated analytical challenges in Heritage Science is widespread. Yet, in recent years, portable instruments developed for in situ analysis have become increasingly available in the market and straightforward to use in heritage studies.^7,12,28,29^ This has clearly come in response to the need for carrying out analyses on location, given strict limitations, in effect by many cultural heritage authorities worldwide, with respect to moving heritage objects to special facilities or collecting samples from works of art and precious objects, monuments, or archaeological findings.

Finally, given the diverse nature of the objects and monuments, as well as the variety of questions that need to be addressed, complementary analytical techniques are often combined because collectively they may provide a more comprehensive characterization of materials and a more global understanding of a specific problem.^30–34^ For example, during archaeological excavations, objects of different nature are uncovered, each requiring a tailored analytical approach to address specific compositional questions. A potential solution to these challenges would involve the use of hybrid instruments that incorporate more than one technique in a single device. However, as of today, hybrid instrumentation has not been commercially available, so several research groups have taken the initiative to design and construct their own prototype instruments.^35–41^

In the context of the present work, we describe a hybrid spectrometer, “TriENA”, literally meaning trident in Greek, that combines three different, well-developed analytical techniques that jointly provide elemental and molecular information: Diffuse Reflectance (DR) spectroscopy, Light-Emitting Diode-Induced Fluorescence (LED-IF) spectroscopy, and Laser-Induced Breakdown Spectroscopy (LIBS). By integrating these methods into a single hybrid instrument, a wider range of materials can be analyzed, extending the spectrometer’s applicability to addressing diverse diagnostic scenarios in the HS field. In the following sections, a brief overview of the basic concept and motivation for building TriENA is given, followed by details on the integrated analytical techniques, the design, and the final engineering approach along with typical examples of data obtained with each technique, which illustrate the performance of the spectrometer.

## Analytical Techniques

The hybrid spectrometer, TriENA, derives its name from the Greek words “tria” (three) and “ena” (one), which denote a combination of three analytical techniques in a single instrument. The system was developed at IESL-FORTH in the context of project CALLOS (Conservation of Athens antiquities with Laser and Lidar technologies Open to Science and public),^42^ a collaborative research effort, coordinated by the Ephorate of Antiquities of the City of Athens (EACA, Ministry of Culture, Greece), which established an *open-to-the-public* conservation laboratory, located in the center of Athens, near the Roman Agora antiquities [37°58′30″N 23°43′21″E]. This is a pioneering conservation workshop aimed at applying innovative laser-based conservation technologies as well as field-deployable and remote-sensing diagnostic instrumentation in the study and conservation of flagship objects and monuments around the Athens archaeological web.

Concentrating on the design of the hybrid spectrometer, we determined three main factors regarding the choice and combination of techniques. First, concerning the analytical perspective, the capacity of the methods chosen to provide valuable and complementary compositional information in a rather straightforward manner was most important. Second, referring to the engineering aspect, component economy (volume/size/weight), and instrument versatility with emphasis on mobility was crucial. In this context, the hybrid instrument’s suite of tools includes LIBS, an atomic spectrometry method, complemented by two molecular spectroscopies: diffuse reflectance (DR) and fluorescence emission spectroscopy based on a light-emitting diode (LED) excitation source (LED-IF). These techniques probe electronic transitions, which are observed across the near-UV, visible, and near-IR part of the electromagnetic spectrum (200–1000 nm). Hence, they share some basic components, including spectrometers and optics, leading to significant cost and size reductions in comparison to the use of three separate instruments. The compact size of the hybrid offers logistic advantages, as well, simplifying transportation and facilitating on-site analysis. A third factor, equally important, for selecting these three techniques, was their suitability to characterize a broad spectrum of materials and optimally address analytical questions in the context of diverse cases of interest investigated by conservators and conservation scientists at the EACA laboratories, taking into consideration the main types of objects and monuments involved in their studies, not only in the context of the CALLOS project but also at a longer-term perspective. For example, a blue LED source emitting at 455 nm was specifically chosen for the LED-IF module, aiming at the identification of epilithic biological attack on stone monuments and buildings.^43^ It is noted, however, that the versatility of the hybrid instrument and the complementary nature of the selected techniques permit its use for the analysis of a diverse range of samples and objects, including metals, paintings, ceramics, and more.

As mentioned in the Introduction section, Raman spectroscopy has been a broadly applied spectrochemical method in the heritage field, providing information about both organic and inorganic materials. As such, a Raman module could be a valid candidate component for TriENA. Nonetheless, taking into consideration known aspects concerning the Raman signal intensity and common interference by fluorescence emissions, it became evident that several features of the hybrid instrument would have to be compromised. Additional detectors and a separate laser source would be needed, increasing the instrument’s complexity and weight. Consequently, in order to preserve portability and suitability for field measurements, the current design does not include a Raman module.

Next, some basic aspects concerning the three techniques, incorporated in the TriENA instrument, will be briefly discussed in the context of the analysis of heritage materials.

### Diffuse Reflectance Spectroscopy

The interaction of light with matter in the ultraviolet (UV), visible (Vis), and near-infrared (NIR) spectral ranges is the basis of common analytical techniques relying on wavelength-specific transmission/absorption or photoluminescence emission. The reflected and/or transmitted light from a sample in these spectral ranges carries information related to the electronic and vibrational energy levels of the molecules or materials comprising the sample. This information is exploited by several spectrochemical techniques that aim to identify the composition of the material/object being analyzed. Considering, more specifically, the analysis of solid, opaque objects, which is a typical scenario in heritage studies, transmittance measurements are no longer possible, and therefore, diffuse reflectance (DR)-based methods are needed in order to monitor the attenuation of incident radiation as a result of light absorption processes. These typically probe transitions between molecular electronic levels of chromophores, or band gap transitions in the case of extended solids, which are observed in the UV, Vis, and NIR^44^ often giving rise to characteristic spectral features. Bands in the NIR may also be originating from overtone or combination vibrational transitions.

Diffuse reflectance spectroscopy across the UV, Vis, and NIR spectral range stands out for its simplicity; hence, it has been widely used, either in single-point analysis or in the form of multi- or hyperspectral imaging studies.^45^ For collecting DR spectra, a broadband white light source can be used in combination with compact spectrometers and regular optics or fiber optics, in relatively inexpensive, compact, and straightforward-to-use instrumentation. Via diffuse reflectance spectroscopy, one effectively probes the color or materials and, because of its simplicity in use and noninvasive character, the technique has been employed in studies of heritage objects including the analysis of pigments and dyes in various objects such as paintings, illuminated manuscripts, walls, ceramics, or textiles.^29,37,46,47^

### LED-Induced Fluorescence Spectroscopy

Absorption of UV or visible light by chromophores results in their electronic excitation, promoting, for example, an electron from occupied to unoccupied molecular orbitals in organic molecules, within the d orbitals in ligand-coordinated metal ions, or from the valence to the conduction band in semiconductor solids. These excitations relax via multiple paths, radiative and/or nonradiative ones, depending on the electronic structure of the chromophore. In the case of relaxation of an excited organic molecule, a photon is typically emitted from the lowest singlet excited state to the vibrational manifold of the singlet ground state. This can be an electronically or vibronically allowed radiative transition, termed fluorescence emission. De-excitation from an excited triplet state to the ground-state singlet is a spin-forbidden transition, and the corresponding emission is referred to as phosphorescence. Fluorescence emission is far more common than phosphorescence in organic molecules, and, indeed, fluorescence spectroscopy has been exploited across many different fields, including biology and biochemistry, environmental monitoring, as well as Heritage Science.^2,39,47–49^ Similar relaxation processes explain the emissions observed in metal complexes, while electron–hole recombination is responsible for emissions observed in semiconductors.

Fluorescence or, more generally, photoluminescence emission spectra are typically obtained by illuminating a sample at a fixed excitation wavelength and recording the intensity of the emitted radiation as a function of the wavelength. This produces a fluorescence emission spectrum. Based on emission bands, characterized by their emission maxima and spectral widths, one can identify different types of chromophores present in a sample. The principles of fluorescence spectroscopy are described in more detail elsewhere.^50^

As regards instrumentation, different implementations are possible based on a) the type of light source used to excite the sample, which can range from broadband white light sources to monochromatic lasers, b) the detector employed (photomultipliers, CCD, etc.), c) the use of monochromators or simple band-pass filters for wavelength selection, and d) the nature of the sample (gas, liquid, solid). In recent years, due to their low cost, size, and reliability, LEDs have been introduced as convenient excitation sources, particularly in relation to compact, mobile instruments, leading to the so-called LED-Induced Fluorescence spectroscopy.^51,52^

### Laser-Induced Breakdown Spectroscopy (LIBS)

This atomic spectrometry technique enables the determination of a material’s elemental composition based on the analysis of the plasma emission produced following the interaction of a laser pulse with the sample.^53–55^ Briefly, when a nanosecond laser pulse is focused onto the surface of a solid, a small amount of material is ablated giving rise to the formation of a transient microplasma reflecting the composition of the sample/object through its characteristic emission spectra. The plasma is rich in excited atoms and ions (as well as molecules, clusters, and molecular fragments), which emit radiation during their relaxation, typically in the spectral range of 200–1000 nm. As the atomic transitions between electronic levels are unique for each element, spectroscopic analysis of the emitted radiation allows one to infer the elemental composition of the analyzed object. LIBS stands out as a straightforward technique that requires no or minimal sample preparation; in principle, it can be applied to any kind of sample, regardless of the material’s conductivity properties, size, or shape, under atmospheric conditions. Multielemental information, including light elements, is obtained in a matter of seconds. It is important to note the intrinsic microsampling feature of LIBS. Each laser pulse ablates a small sample volume, confined in a spot of roughly 150 μm in diameter and depth on the order of 1 μm or less. As a result, LIBS is classified as a microinvasive technique.

The simplicity of the key components included in a LIBS setup, mainly a pulsed-laser source for excitation, small-size spectrometers for plasma emission detection, and basic optics for laser beam focusing and plasma emission collection, facilitates the design of compact, robust, and portable instruments suitable for performing field (in situ) measurements. Given its microsampling capacity, LIBS can be exploited for obtaining stratigraphic information, i.e., to study the depth-resolved distribution of elements in a given spot on the object analyzed. This is a crucial capability when investigating multilayered materials such as paintings (varnish-pigment-primer) or corrosion layers. A small amount of material is ablated after each laser pulse is delivered on target, and therefore, collecting spectra with a few successive laser pulses provides a good view of the compositional changes with depth. Capitalizing on these features, LIBS applications in heritage studies have been growing steadily over recent decades, targeting the analysis of diverse objects and in various contexts. Typical cases include identification of pigments in paintings or icons,^15,56^ wall paintings and plaster,^57,58^ or stone monuments,^39,59^ analysis and classification of ceramics and pottery,^60–62^ or metal alloys,^63–65^ analysis of bones and teeth of archaeological interest,^66–68^ paleoclimate studies,^69–71^ etc. For further references, interested readers may consult relevant reviews and book chapters focusing on LIBS applications in cultural heritage.^5,15–17,55,72^

## Experimental Section

The hybrid spectrometer was designed with the purpose of integrating the DR, LED-IF, and LIBS modules into a single instrument on the basis of two main concepts. First, as noted in the previous section, each individual technique produces different information but complementary to what the other two provide; thus, the combined use of the data obtained expands the applicability of the hybrid system to diverse scenarios. Second, the selected techniques share some components and as such make possible the design of a smaller and more compact instrument. Notably, the same spectrometer can be employed for both LED-IF and DR spectroscopy. In addition, the system design ensures that all light beams converge onto the same spatial coordinates; i.e., if the system and the object under study remain stationary, the area probed is identical for all three modules. As a result, light reflected and emitted from the analyzed object originates from the same position on its surface, thus collection optics and fibers can be shared across the different techniques. The latter is possible because, for all three techniques, light is detected across wavelengths in the UV–Vis–NIR range, from 200 to 1000 nm. With its overall operational design permitting a straightforward switch between techniques via customized software, the TriENA hybrid allows researchers to select the most suitable method for each specific case or, if needed, analyze the same area/point on the object surface with all three modules within seconds to a few minutes.

As shown in Figure 1, the instrument consists of two main parts: an optical head and a control unit. The optical head contains all the light sources, focusing/collection optical components, and a viewing camera. The control unit contains three spectrometers, appropriate electronic components, and the power supply units associated with the different light sources. Both units are joined via an umbilical cable, which is detachable, facilitating packaging of the instrument and transportation. The umbilical encloses USB, electrical, and optical fiber cables.

**Figure 1:**
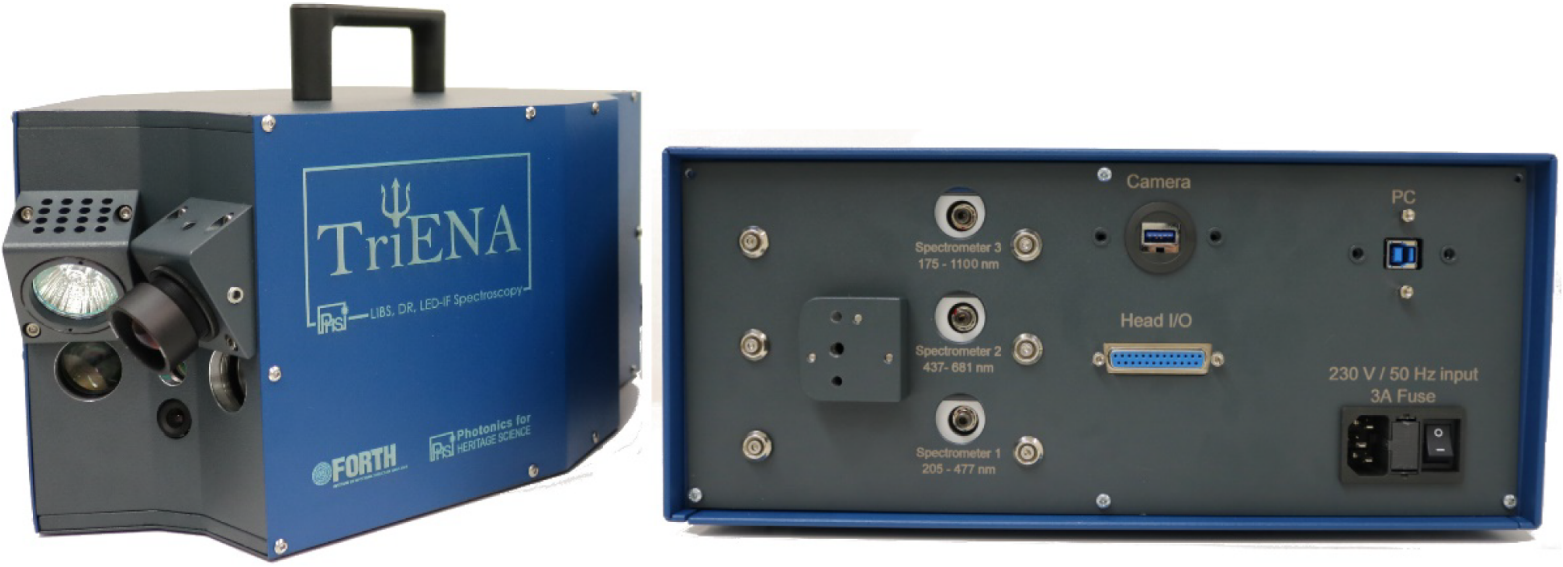
Main units of the portable hybrid system. The optical head (left) houses, in an appropriate geometry, light sources and optics. Of the six different ports seen, in the front face of the head, three correspond to light delivery (white light for DR, LED for LED-IF, and laser for LIBS), two are spectra collection ports, and one is the viewing camera port (see also Figure 2). The control unit (right) contains the spectrometers, power supplies, and electronics. The two units are connected via an umbilical (not shown here) which houses optical fibers, USB, and electrical cables providing also protection.

### Optical Head

A simplified schematic of the optical head is shown in Figure 2, which displays the main components and their arrangement. For clarity, some of the mechanical parts, such as mounts and supporting brackets, laser heatsink, electronic board, USB, and power cables, have been omitted. The compact optical head, measuring approximately 35 × 20 × 15 cm^3^ and weighing approximately 7 kg, is designed so as to be conveniently mounted on top of a tripod or integrated into a customized XYZ stage system, allowing precise and controlled positioning relative to the object under investigation. The LIBS laser (Figure 2a) is placed at the center of the optical head. The output laser beam (λ = 1574 nm) passes through a beam expander (magnification ×3) before being focused onto the object’s surface by means of a plano-convex lens (*f* = +75 mm). This optical configuration yields a spot size of approximately 200 μm in diameter, as determined via test measurements performed on a metal aluminum plate following irradiation by 20 laser shots. Prior to entering the beam expander, the laser passes through a long-pass dichroic mirror (cut-on wavelength: 650 nm). This mirror is in fact used to align an aiming red diode pointer (Figure 2f) along the optical axis of the LIBS laser beam, and consequentially, it can be used to pinpoint the analysis position on the object surface. A viewing camera placed below the laser beam expander (Figure 2e) provides a live video of the object under analysis with a field of view (FOV) spanning 90 × 65 mm^2^ at a working distance (WD) of 70 mm. The camera is oriented at a slight angle, 15° relative to the laser axis, minimizing distortions in the captured images. This tilt together with the aiming diode allows for precise adjustment of the working distance between the optical head and the object investigated. Only when the object’s surface is positioned accurately at the designated WD, the red diode spot and a crosshair displayed in the camera livestream are coincident right at the point where analysis is to be performed. Any deviation from this position causes the diode and crosshair to diverge, providing real-time feedback for correcting object placement.

**Figure 2:**
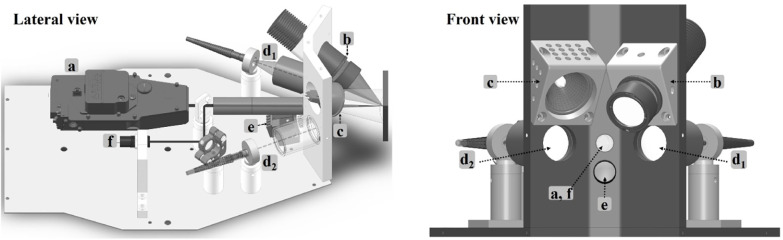
Simplified schematic of the optical head, lateral view (left), and front view (right), displaying the main components: LIBS laser source (a), LED-IF source (b), quartz tungsten-halogen lamp (c), collection optics assemblies (d_1_,d_2_), viewing camera (e), and aiming red diode pointer (f).

For the diffuse reflectance measurements, a quartz tungsten-halogen (QTH) lamp (see Figure 2c), with a color temperature, *T*_color_ ≈ 2800 K, is positioned approximately at 40° relative to the laser axis at the upper part of the optical head. A heatsink is employed to efficiently dissipate the heat generated (see Figure 2c, shown only in the front view). The LED source employed in the fluorescence measurements is symmetrically situated on the upper section of the optical head, positioned opposite to the QTH lamp. Since this hybrid instrument was developed in the framework of the CALLOS project and one of the main tasks of this project was the analysis and identification of biodeterioration on archaeological stone surfaces, a LED source emitting at 455 nm was selected based on results obtained in a previous investigation by our group.^43^ An aspheric condenser lens (*f* = +20 mm) focuses the LED emission onto the analyzed object; in addition, a band-pass filter is used to reduce the spectral width of the LED emission (FWHM = 10 nm). It is noted that the design and orientation of the three sources (laser, LED, and QTH lamp) are such that their light beams converge at the same point. This deliberate arrangement facilitates rapid sequential analysis of the same object area via all three techniques, enabling a comprehensive analysis of the object in a matter of a few minutes. Finally, the optical head incorporates two optical collection assemblies placed on either side of the laser axis. One of them (Figure 2d_1_), fitted with UV-grade lenses, is optimized for collecting light in the spectral region 200–400 nm, while the other (Figure 2d_2_) is equipped with achromatic lenses and optimized for the spectral range 400–1000 nm. Both optical collection assemblies focus the gathered light into corresponding optical fibers connected with the spectrometers included in the control unit (see the next section for more details). While LIBS spatial resolution is inherently determined by the focused laser spot size (∼200 μm diameter), the resolution of the DR and LED-IF modules is governed, in the present instrument, by the field of view of the optical collection arrangement rather than by the dimensions of the areas illuminated by the corresponding light sources. In fact, the areas illuminated by the LED and the QTH lamp, correspond to diameters of approximately 5 mm and several cm, respectively. However, the diameter of the area probed by the collection systems is confined to approximately 1 mm. As a results, only light coming from that 1 mm diameter spot is collected by the lens system defining the spatial resolution of the LED-IF and DR modules in the instrument setup. Table 1 provides a comprehensive overview of technical details concerning the main components housed in the optical head.

**Table 1:** Technical Details of the Main Components in the Optical Head

Component	Technical details
LIBS laser [a]^a^	Falcon (Lumibird), diode pumped, Q-switched OPO Nd:YAG. λ = 1574 nm (eye safe), *E*_pulse_ = 3.85 mJ (at the sample), τ_pulse_ ≈ 6 ns, *f*_pulse_: single shot to <40 pulses/s, beam diameter *d* = 1.2 mm
LED-IF source [b]	LED M455L4 (Thorlabs). λ = 455 nm, Δλ_FWHM_ ≈ 10 nm (after band-pass filter), *P* = 30 mW (at the sample)
DR source [c]	Quartz tungsten-halogen (QTH) lamp. *P* = 10 W, *T*_color_ ≈ 2800 K
Viewing Camera [e]	Alvium 1800 U-500C (Allied Vision). CMOS color sensor, 2592 × 1944 pixels, frame rate = 68 fps, WD ∼70 mm, FOV 90 × 65 mm^2^
Aiming diode pointer [f]	D08ACCTLST red diode (Optris) λ = 635 nm
UV collection assembly [d1]	×2 UVFS plano-convex lenses, Diameter *D* = 1″, focal lengths: *f*_1_ = +50 mm, *f*_2_ = +80 mm. Optical fiber: core diameter = 600 μm, numerical aperture (NA) = 0.22, Length = 2 m; Solarization resistant
VIS-IR collection assembly [d2]	×2 achromatic doublets, Diameter *D* = 1″, focal lengths: *f*_1_ = +50 mm, *f*_2_ = +80 mm. Bifurcated optical fiber: core diameter = 600 μm, numerical aperture (NA) = 0.39, Length = 2 m

a Brackets denote components as labeled in Figure 2.

### Control Unit

As briefly noted, the control unit box (dimensions 45 × 35 × 14 cm^3^, weight ∼10 kg) houses three compact spectrometers, the power supplies for the different light sources, an industrial-grade USB hub, and different electronic components (Figure 1, right). It provides all necessary interfacing to the head and the laptop computer (PC) used to control the spectrometer operation and data acquisition.

Two of the compact spectrometers (see Table 2 for details) are dedicated to LIBS data acquisition at a medium-high spectral resolution (0.3 nm) and cover the UV and visible spectral ranges, 207–475 nm and 439–680 nm, respectively. In this range, the majority of emission lines, typically detected in LIBS measurements, can be observed. The third spectrometer is shared by both the LED-IF and the DR modules and covers a wider spectral range (175–1100 nm) at a lower spectral resolution (2.2 nm), yet adequate for acquiring reflectance and fluorescence emission spectra. All three spectrometers along with the viewing camera are connected to an industrial-type USB3 hub, which in turn is connected to a laptop computer (PC) used to control the hybrid instrument. Technical details of the different components included in the control unit are listed in Table 2.

**Table 2:** Technical Details of the Main Components Included in the Control Unit

Component	Technical details
LIBS Spectrometers	x2 Fiber optic spectrometers, AvaSpec-ULS4096CL-EVO (Avantes) 75 mm AvaBench. 4096 pixel CMOS detector, grating: 1200 lines/mm, entrance slit: 25 μm, Resolution: Δλ = 0.3 nm. Spectral range: Spec. 1 (207–475 nm). Spec. 2 (439–680 nm).
LED-IF and DR spectrometer	Fiber optic spectrometer AvaSpec-ULS2048CL-EVO (Avantes) 75 mm AvaBench. 2048 pixel CMOS detector, grating: 1300 lines/mm, entrance slit: 50 μm, Resolution: Δλ = 2.2 nm. Spectral range: Spec. 3 (175–1100 nm).
USB3 hub	USB3 Hub ADVANTECH BB-USH207

The control unit interfaces with the optical head by means of a detachable umbilical that accommodates and protects the USB3 camera cable and the Head I/O cable, which is used to supply with power the light sources in the head and facilitate communication between the system laptop and the laser head electronics. Additionally, the umbilical encloses the two optical fibers that couple light collected by the two optical collection ports, located at the front of the head, into the three spectrometers via SMA inputs (with one of the optical fibers being a 2-to-1 bifurcated bundle). To facilitate convenient transportation of the instrument for on-site campaign measurements, the umbilical tube is easily detached from the back of the control unit. This allows the instrument to be packed into two individual carrying cases, each with a manageable weight of approximately 10 kg.

Spectrometer control and spectral data acquisition are performed using AvaSoft, the Avantes proprietary software. Acquisition parameters including integration time (τ_G_) in DR and LED-IF measurements as well as the integration time (τ_G_) and integration delay (τ_DELAY_) during LIBS measurements are controlled via this software. The light sources (laser, QTH, and LED) are controlled and operated via a customized LabVIEW application, also displaying the live feed from the viewing camera, which helps the operator select the position on the sample/object where analysis is to be carried out. In addition, images of the object can be saved during the analysis. A screenshot from the laptop PC displaying the whole layout of the interface software is shown in Figure 3.

**Figure 3:**
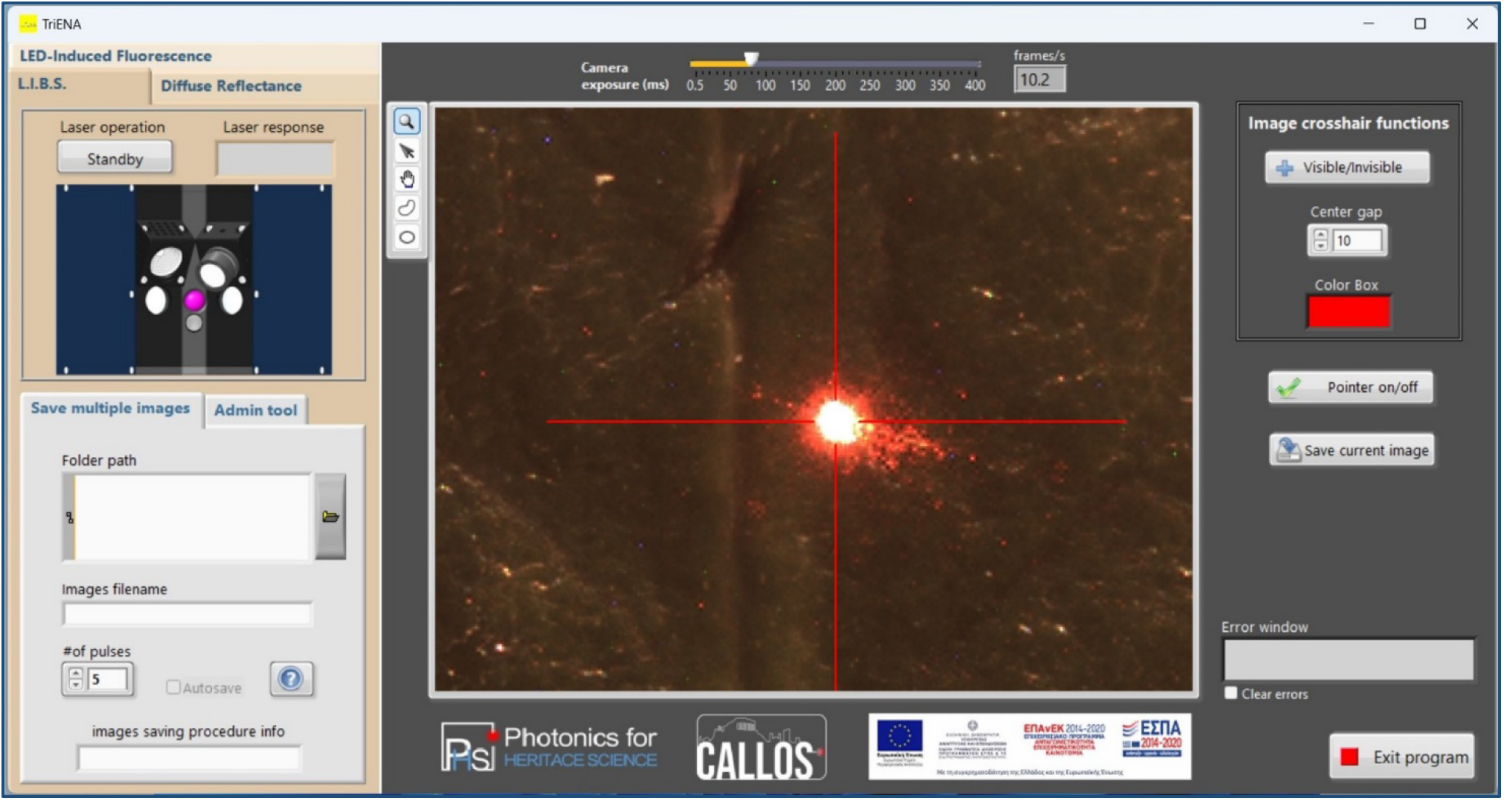
Screenshot of the custom software interface. When the red pointer is at the center of the image crosshair (as shown), the object under analysis is located at the optimum working distance. The software controls all light sources (laser, LED, and QTH lamp) and the viewing camera.

## Results and Discussion

### Diffuse Reflectance Analysis

A diffuse reflectance measurement probes the efficiency of a rough, opaque surface to reflect (backscatter) an incident probe beam. This is expressed as the ratio of the light intensity reflected by the sample over the intensity reflected by a reference surface, nonabsorbing at the wavelength range of interest. If reflectance measurements are collected for different wavelengths, a spectrum is obtained, which can be represented and computed by using eq 1:

1 $$R\left(\lambda \right)=\frac{I_{S}\left(\lambda \right)-I_{D}\left(\lambda \right)}{I_{ref}\left(\lambda \right)-I_{D}\left(\lambda \right)}$$

*R*(λ) represents the diffuse reflectance spectrum, while *I*_S_(λ) is the intensity of the light reflected by the sample surface (S) at a given wavelength, λ, and *I*_ref_(λ) denotes the corresponding signal obtained from a “white” reference standard, namely, a surface that approaches the ideal white (nonabsorbing) behavior exhibiting unity reflectance across the spectral range of interest. *I*_D_(λ) is the signal recorded by the detector when the sample is not illuminated (lamp off) and the object remains at the analysis position; thereby, *I*_D_ accounts for any contribution coming from ambient stray light (often referred to as the dark signal).

With the above equation in mind, the procedure for carrying out a DR measurement begins with the acquisition of a dark signal, *I*_D_(λ), and next of the reference spectrum, *I*_ref_(λ). To obtain the latter, the QTH lamp illuminates a white diffuse reflectance standard (Spectralon, Labsphere), known for its spectrally flat reflectance in the Vis and NIR. The standard is set as accurately as possible at the position where the actual object under analysis is going to be placed, and the reference spectrum is then recorded on the broadband spectrometer, Spec. 3. To speed up this step, the reflectance standard has been attached onto a plug-and-play customized bracket (Figure 4, right), which can be easily attached to (and detached from) the optical head, ensuring that its surface remains at the optimum working distance, considerably reducing the time needed for each measurement. Furthermore, this arrangement maintains both the reference standard and the actual sample in, as much as possible, identical orientation with respect to the incident beam and the angle of observation (line of sight) defined by the position of the collection optics. In this way, variations due to Lambert’s emission law are minimized.

**Figure 4:**
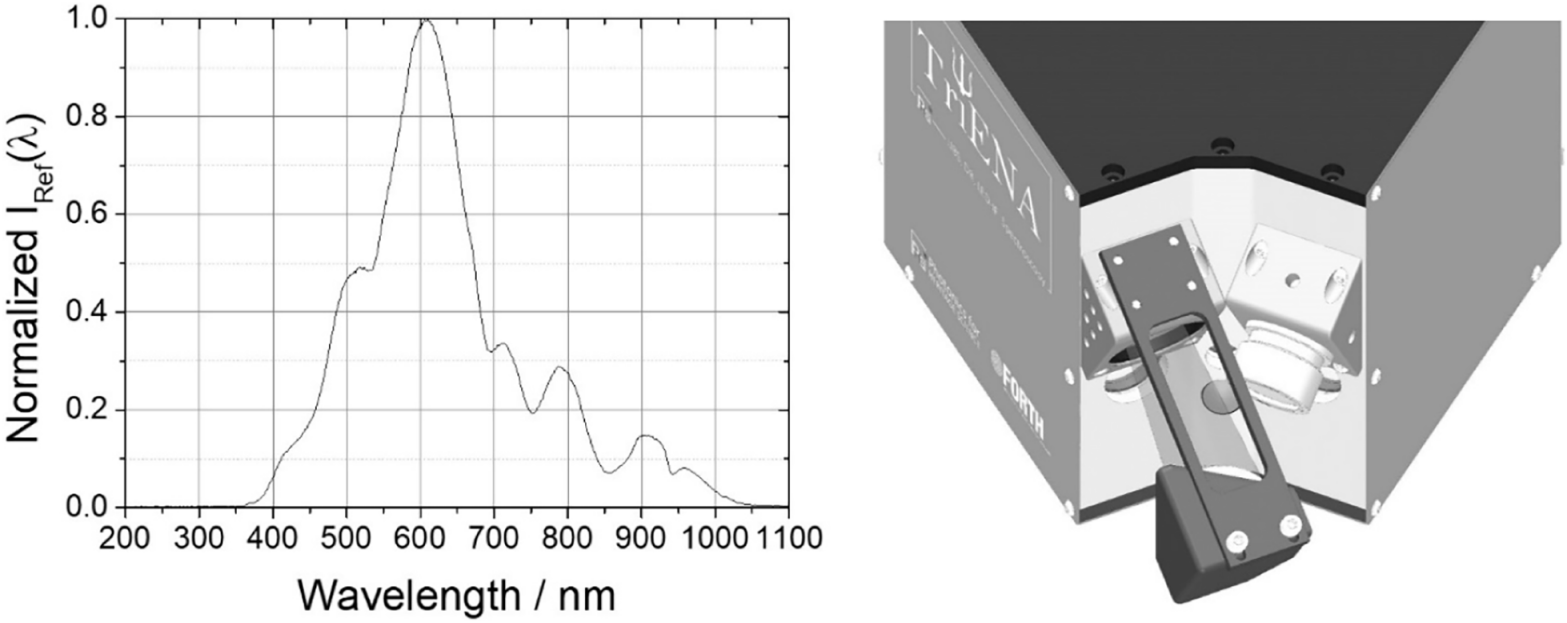
(left) Normalized emission intensity, *I*_ref_(λ), of the QTH lamp as recorded by the system (τ_G_ = 3 ms) on a white diffuse reflectance standard. The intensity profile reflects the spectral sensitivity of the spectrometer. The resultant effective spectral range extends from approximately 400 to 1000 nm. (right) Top view of the optical head indicating the plug-and-play bracket that holds the reference standard, attached to the lamp heatsink element (Locking and unlocking the bracket in place can be done in a matter of seconds).

A typical reference spectrum, acquired by employing an integration time of τ_G_ = 3 ms, is displayed in Figure 4 (left). Under these experimental conditions, the operational spectral range spans from approximately 400 to 1000 nm. It is important to note that, if necessary, a longer integration time can be employed to extend the spectral range by about 25–30 nm on either side of this range. However, an increase of τ_G_ leads to signal saturation in the central part of the spectrum, and in such a case, a separate measurement would be necessary for obtaining accurate reflectance information from the extended spectral range. Commenting on the profile of *I*_ref_(λ), this represents the convolution of the QTH lamp output (nearly blackbody, corresponding to temperature *T*_color_ ≈ 2800 K) with mainly the instrument response function of Spec. 3, which in turn is determined by the efficiency of the diffraction grating and the sensitivity of the CMOS sensor across the observed spectral range.

To assess the performance of the DR module, different painting mock-up samples were examined (Figure 5a). The first one consisted of two tempera paint strips (red and yellow pigments dispersed in an egg yolk binder) applied over a gesso preparation layer (gypsum with animal glue) on a wood substrate. The second mock-up represented a green fresco painting made with malachite pigment over a calcium carbonate substrate. The corresponding DR spectra are shown in Figure 5b, along with reference DR spectra of the corresponding pure pigments obtained by using a benchtop laboratory instrument equipped with an integration sphere (PerkinElmer Lambda 950, PE). These reference pigments include cadmium red (CdS_*x*_Se_1–*x*_, Kremer 21130), yellow ochre (FeO­(OH), Kremer 48000), and malachite (CuCO_3_·Cu­(OH)_2_, Kremer 10300). The small spectral differences between the mock-up paints and the corresponding neat pigment samples are attributed to the fact that, in the paint samples, pigments were mixed with a medium, unlike the neat pigments, which were tested in the form of a pressed powder pellet. Nevertheless, the main spectral features of each pigment, such as band center and width, remain identifiable, providing valuable information for the characterization of the pigments based on their DR spectra. An additional feature that can facilitate intercomparison and interpretation of spectral data relies on the use of first derivative spectra, d*R*(λ)/dλ. These spectra vanish at band maxima or minima of the DR spectrum while showing peaks at its inflection points (Figure 5c). As such, they can accentuate features that may not be strong in the normal *R*(λ) spectra.

**Figure 5:**
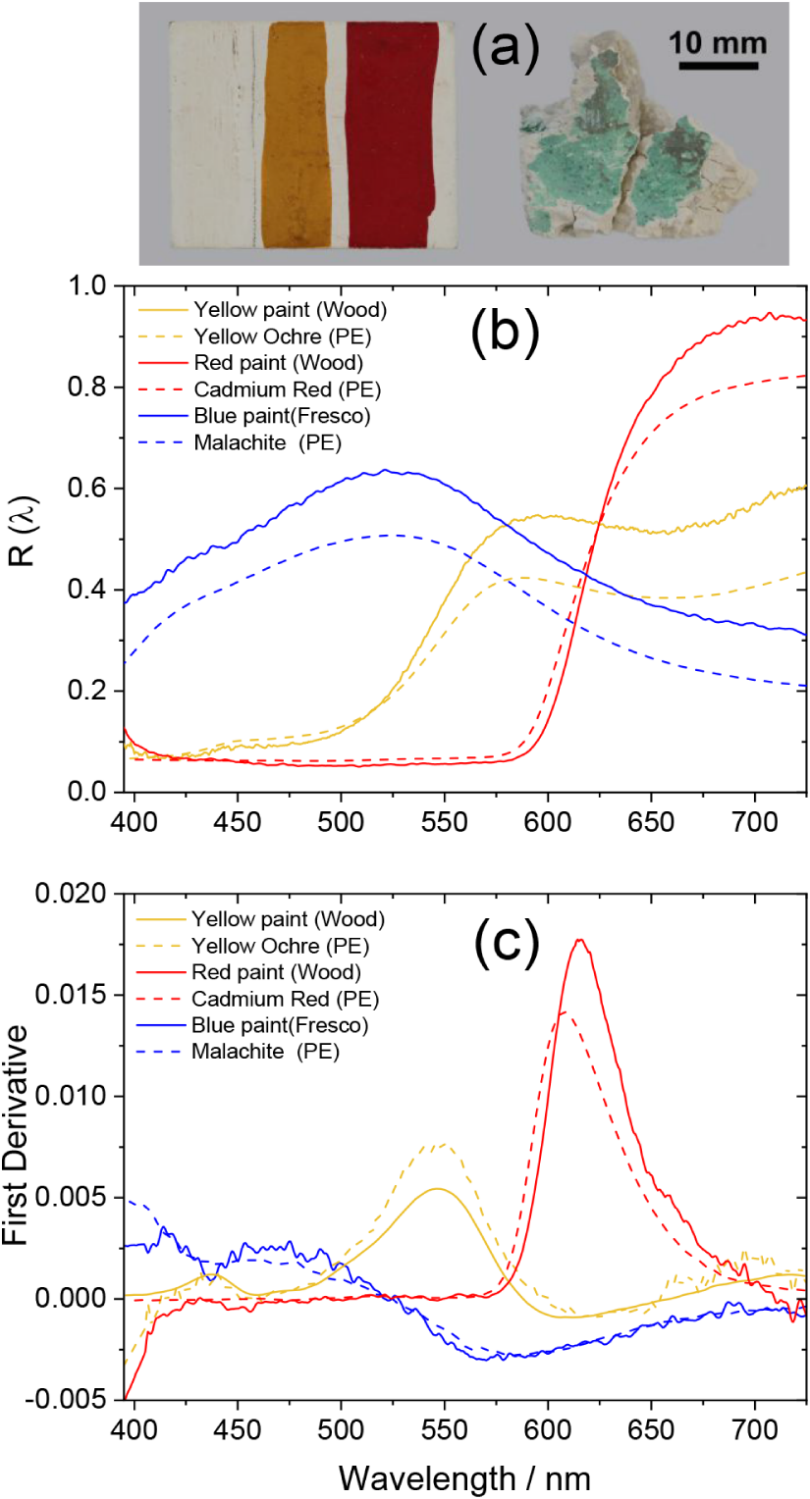
(a) Test paint samples. (b) Diffuse reflectance spectra, *R*(λ) (solid lines), of red, yellow, and blue pigments obtained using the DR module of the hybrid spectrometer (τ_G_ = 3 ms); reference spectra recorded on a PerkinElmer (PE) Lambda 950 laboratory benchtop absorbance/reflectance spectrophotometer are displayed for comparison purposes (dashed lines). (c) First derivative of the diffuse reflectance spectra.

A rather more challenging case is illustrated in the data shown in Figure 6. A mock-up blue fresco painting made with Egyptian blue (EB) pigment on a carbonate substrate was examined, and its DR spectrum was found, not surprisingly, to exhibit high average reflectance, given the very light hue of the paint. Nevertheless, relatively weak spectral features are observed, which in fact correspond to EB, as verified by comparison with the reflectance spectrum collected from the neat pigment (CaCuSi_4_O_10_, Kremer 10060, particle size <120 μm). This case points out to limitations arising when reflectance spectra are obtained from paints containing low amounts of pigment material dispersed in a white medium or mixtures of pigments. In this context, a complementary tool for analysis would provide further evidence with respect to the identity of a paint as shown in the following section, where the analysis of the same paint is described, performed by use of LED-IF spectroscopy.

**Figure 6:**
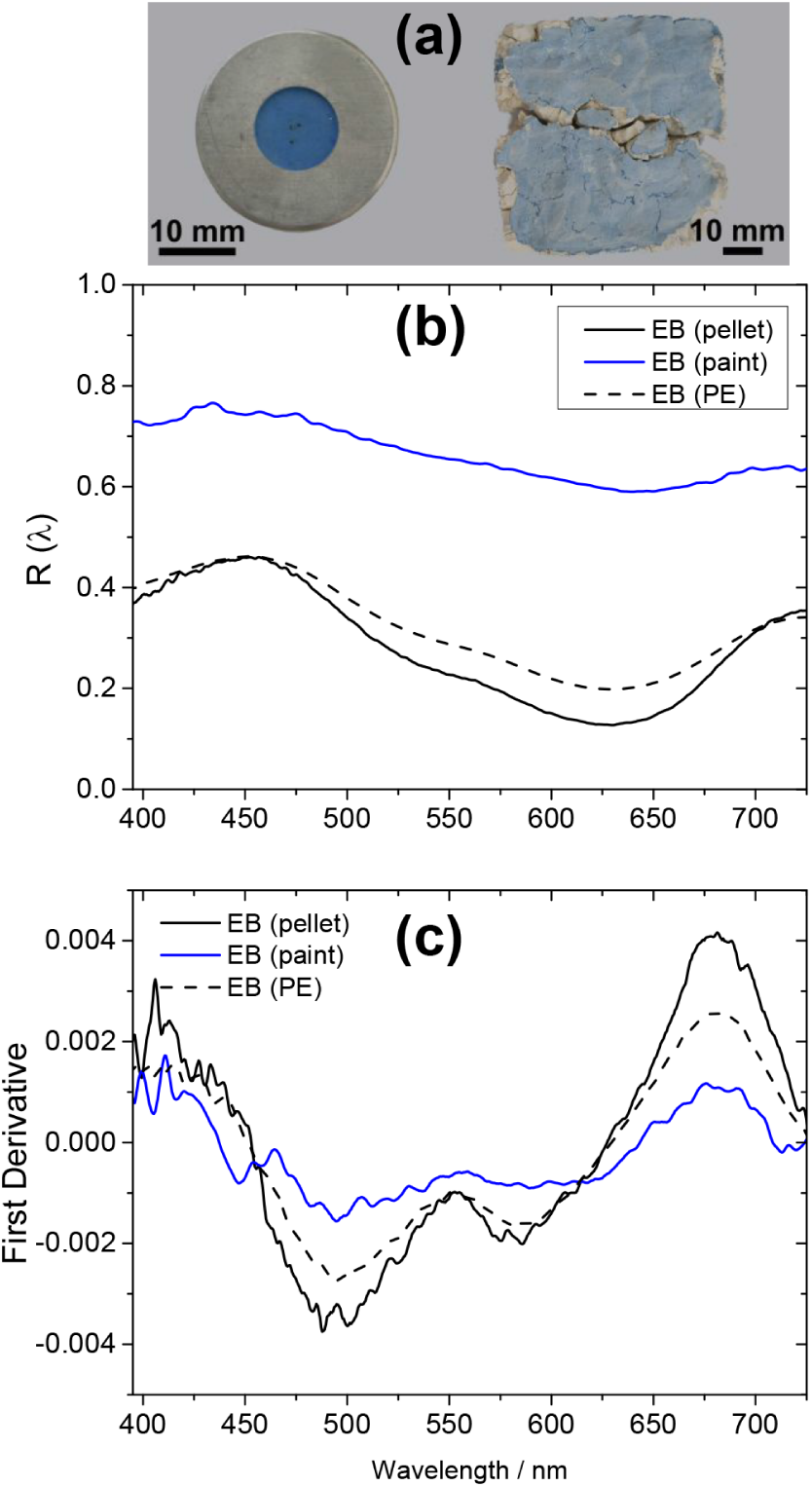
(a) Test paint samples. (b) Comparison of DR spectra of Egyptian blue pigment in different forms: neat as a pressed pellet and as a mock-up fresco painting (τ_G_ = 3 ms). A reference spectrum of neat Egyptian blue pigment, acquired using a PerkinElmer (PE) Lambda 950 laboratory benchtop absorbance/reflectance spectrophotometer, is shown for comparison. (c) First derivative of the diffuse reflectance spectra.

### LED-Induced Fluorescence Analysis

Typically, the procedure for acquiring a fluorescence spectrum with the LED-IF module of the TriENA system involves irradiation of the sample surface with the LED source and collection of the emission in backscattering mode via the lens into the broadband spectrometer, Spec. 3. Acquisition time, τ_G_, in the range of a few tens of milliseconds gives rise to good-quality spectral data, displayed as intensity of fluorescence emission versus wavelength, *I*_F_(λ). Longer values of τ_G_ are required when materials with a low fluorescence yield are examined.

Continuing from the previous section, Egyptian blue is known to exhibit characteristic fluorescence emission, with a band maximum at around 900 nm, well documented in the literature and widely exploited by heritage scientists and conservators for the identification of this pigment in various objects and monuments.^73,74^ In this context, LED-IF was employed in order to obtain additional information, complementary to that collected via DR analysis. Given its absorbance spectrum (see Figure 6), EB is optimally excited with light in the range of 550–650 nm. Despite the fact that the LED excitation source has its maximum emission at 445 nm, which is not ideal for exciting EB, the emission spectra collected from both neat EB and the EB paint sample (Figure 7) show clearly the characteristic emission band of the pigment in the NIR region, enabling its unambiguous identification. It is further noted that the versatility of the system and its design permit the user to bypass the integrated LED emitting at 455 nm and use instead an external light source, for example, a laser pointer, if that provides more appropriate excitation. Figure 7 includes the fluorescence spectrum of the wall-painting sample, excited by a standard red diode laser pointer emitting at 650 nm (as seen in the saturated feature in the spectrum), in which the characteristic EB emission band is significantly enhanced. Adding to this, it should be noted that the mechanical design of the optical head allows, if necessary, quick replacement of the Thorlabs 455 nm LED with any LED from the multiple wavelength options available within the same vendor’s series, for example, one emitting in the UV.

**Figure 7:**
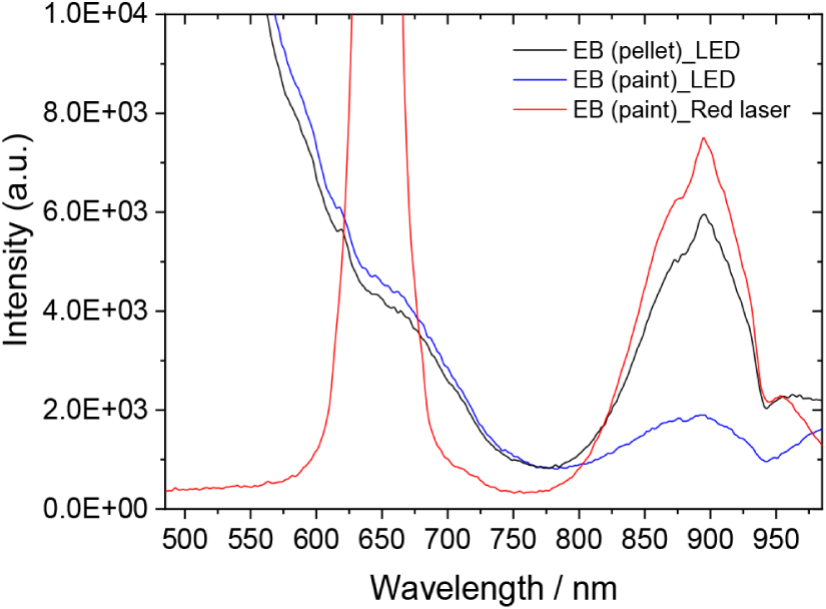
Fluorescence emission spectra of Egyptian blue, in neat form (as a pressed pellet) and as a mock-up fresco painting applied over a carbonate substrate. Excitation: λ = 445 nm (LED), 650 nm (external laser pointer). Acquisition: τ_G_ = 100 ms.

As noted in the Analytical Techniques section, one of the key objectives of the CALLOS project was to come up with a tool capable of identifying and classifying environmental (pollutant) deposits on stone monuments and buildings. This type of information aids conservators in deciding on the specific cleaning approach they might need to apply in each case. Fluorescence emission, and in particular LED-IF spectroscopy, was selected as a tool known for its ability to detect and discriminate among various types of biodeterioration on stone surfaces. With this in mind, extensive work has been carried out^43^ addressing a 2-fold goal: a) to develop a proper analytical methodology for classifying biological deposits on stone based on their characteristic fluorescence emissions and b) to identify the optimum components to be integrated in the LED-IF module of the hybrid, including the selection of the excitation wavelength. In order to illustrate the performance of the integrated LED-IF module and compare it with our previously used laboratory setup, a series of tests were performed on samples from stone monuments bearing deposits of biological origin. Figure 8 displays typical LED-IF spectra from a stone sample with evident biological crust (Object B, see ref 43) and from a green leaf of a Hibiscus plant. The latter shows, as expected, two strong emission bands from chlorophyll species at 685 and 735 nm, respectively. The emission collected from the stone sample is comparable to that reported in our previous work,^43^ with a main band at 660 nm indicating the presence of allophycocyanin, a pigment characteristic of cyanobacteria present in lichens.

**Figure 8:**
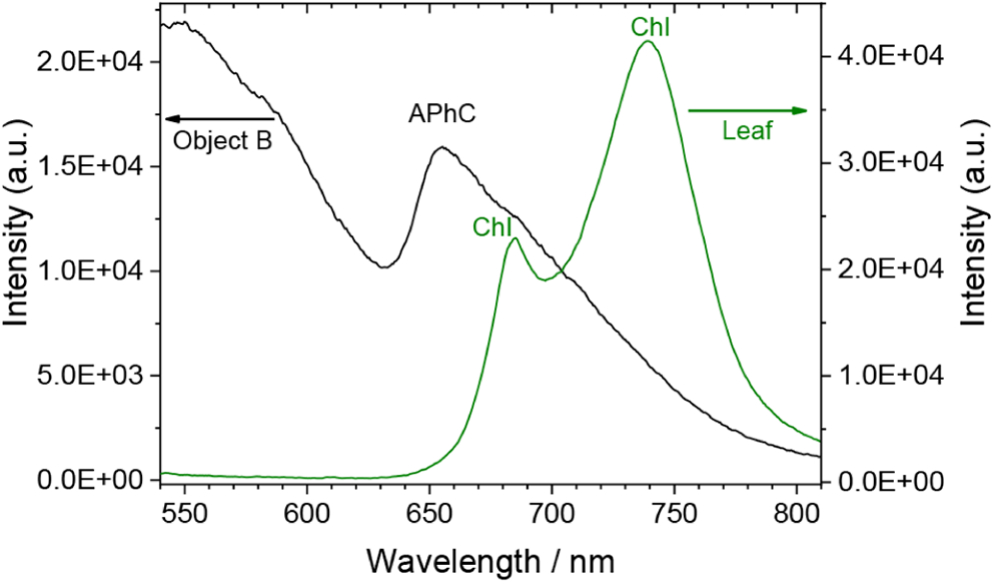
LED-IF emission spectra from a stone bearing a biological deposit and a Hibiscus leaf. Excitation: λ = 445 nm (LED). Acquisition: τ_G_ = 50 ms.

From the technical standpoint, it is worthwhile noting that the integration time employed for the leaf measurement was τ_G_ = 50 ms, approximately 6 times shorter than that used in similar measurements conducted during our preliminary studies, confirming the enhanced performance of the LED-IF setup in the new instrument. While this reduction in τ_G_ may not affect significantly the speed of analysis (given that acquisition time is negligible compared to the time required for setting up the experiment, i.e., selecting analysis points and setting the optimum working distance), it is important to note that minimizing the integration time not only reduces the exposure of the object to LED light, thereby lowering the risk of photodegradation in sensitive samples, but also minimizes interferences on the recorded spectrum arising from the ambient light. The latter effect becomes particularly relevant when measurements do not take place in a controlled environment (laboratory) but on-site, where excluding ambient light is not possible.

### Laser-Induced Breakdown Spectroscopy Analysis

A LIBS measurement involves the irradiation of the sample/object surface by a single pulse from the laser source, leading to plasma formation right at the spot targeted for analysis. The emission from the plasma is collected by the UV and Vis lenses and fiber-optically relayed into the Spec. 1 and Spec. 2 spectrometers, respectively. Each such measurement yields the corresponding plasma emission spectrum, *I*_LIBS_(λ), for example, the one displayed in Figure 9, which was obtained during the analysis of a metal sample simulating an archaeological ternary bronze alloy (w/w composition: 90.6% Cu, 3.4% Sn, 3.9% Zn, 2.0% Pb). The LIBS spectrum shown reflects the elemental composition of the alloy and has been the result of a single laser-pulse measurement carried out at a point of interest in the metal alloy surface with no requirement for any sample or surface preparation. The targeting of the analysis point, as well as the positioning of the metal object at the optimum working distance, namely, at the focal spot of the laser focusing lens, is facilitated by the combination of the viewing camera image crosshair and the pointer. Total analysis time, including sample positioning and spectrum acquisition, is typically on the order of a minute.

**Figure 9:**
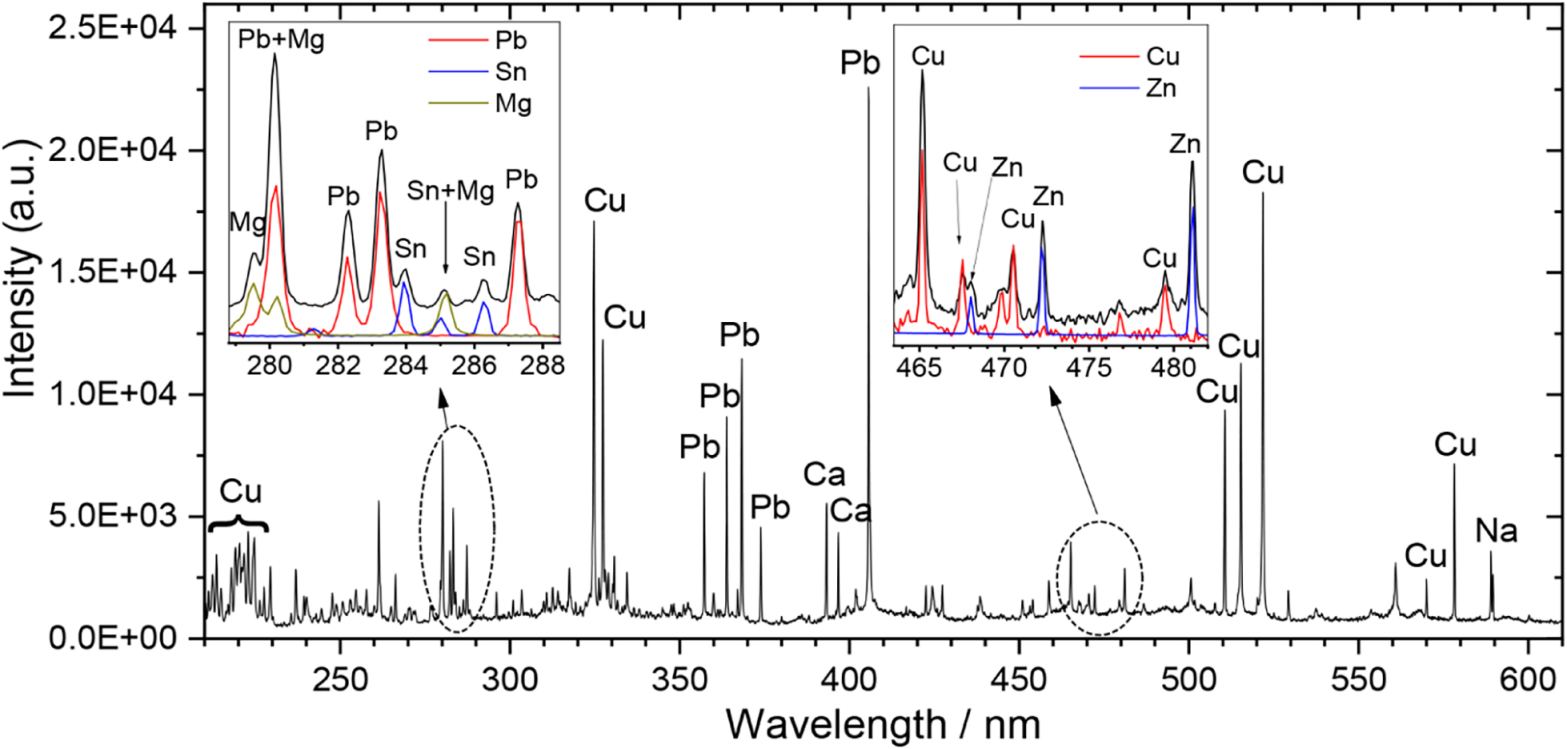
Single-shot LIBS spectrum of a Cu–Sn–Zn–Pb bronze alloy (τ_DELAY_ = 1 μs, τ_G_ = 30 μs). Emission lines of Cu are dominant in the spectrum, but multiple peaks from other alloy elements (Zn, Pb, and Sn) are also clearly identified. The spectrum also exhibits emission lines originating from surface pollutants such as Ca, Mg, and Na. Insets: Selected parts of the spectrum overlaid with corresponding LIBS spectra from high-purity metal samples (Pb, Sn, Mg, Cu, Zn) verify peak assignment.

The system exhibits a good response across the whole spectral range covered, and peaks with a good signal-to-noise ratio (S/N) are detected even at wavelengths below 225 nm. This fact, combined with the resolution of the spectrometers, facilitates multielemental analysis of the object. In the specific case discussed (Figure 9), the majority of the spectral lines are attributed to the major alloy element, Cu, while multiple emissions from the rest of the alloy elements, Sn, Zn, Pb, and surface pollutants like Na and Ca, are easily identified in the spectrum.

A common application of LIBS in the Heritage Science field concerns the identification of pigments in painted works of art. To illustrate the performance of the LIBS module in these types of scenarios, the red paint, also examined by DR (Figure 5), was analyzed. Confirming the findings of the DR analysis, the single-shot LIBS shown in Figure 10 proves the presence of cadmium on the basis of several spectral peaks attributed to cadmium emissions. Cadmium red is a sulfoselenide; hence, both Se and S emissions should ideally have been identified in the LIBS spectrum, but this was not the case. It should be noted that the most intense Se I emission lines (196.09 and 203.98 nm) fall outside the spectral range of the spectrometer. Likewise, the most intense emission lines of sulfur (S I 180.731, 182.624, and 921.286 nm) fall in the VUV and NIR, respectively, both outside the range of the current spectrometer. Despite that, combining LIBS with DR or LED-IF, if necessary, can provide reliable pigment identification when sulfur- or selenium-containing pigments or fillers are suspected. Other, less intense peaks in the LIBS spectrum are assigned to elements such as Ca, Mg, Ba, and Na, which are compatible with pigment impurities and pollutants on the mock-up surface. A table of the labeled peaks in the LIBS spectra of Figures 9 and 10, with their spectroscopic information, is provided in the Supporting Information.

**Figure 10:**
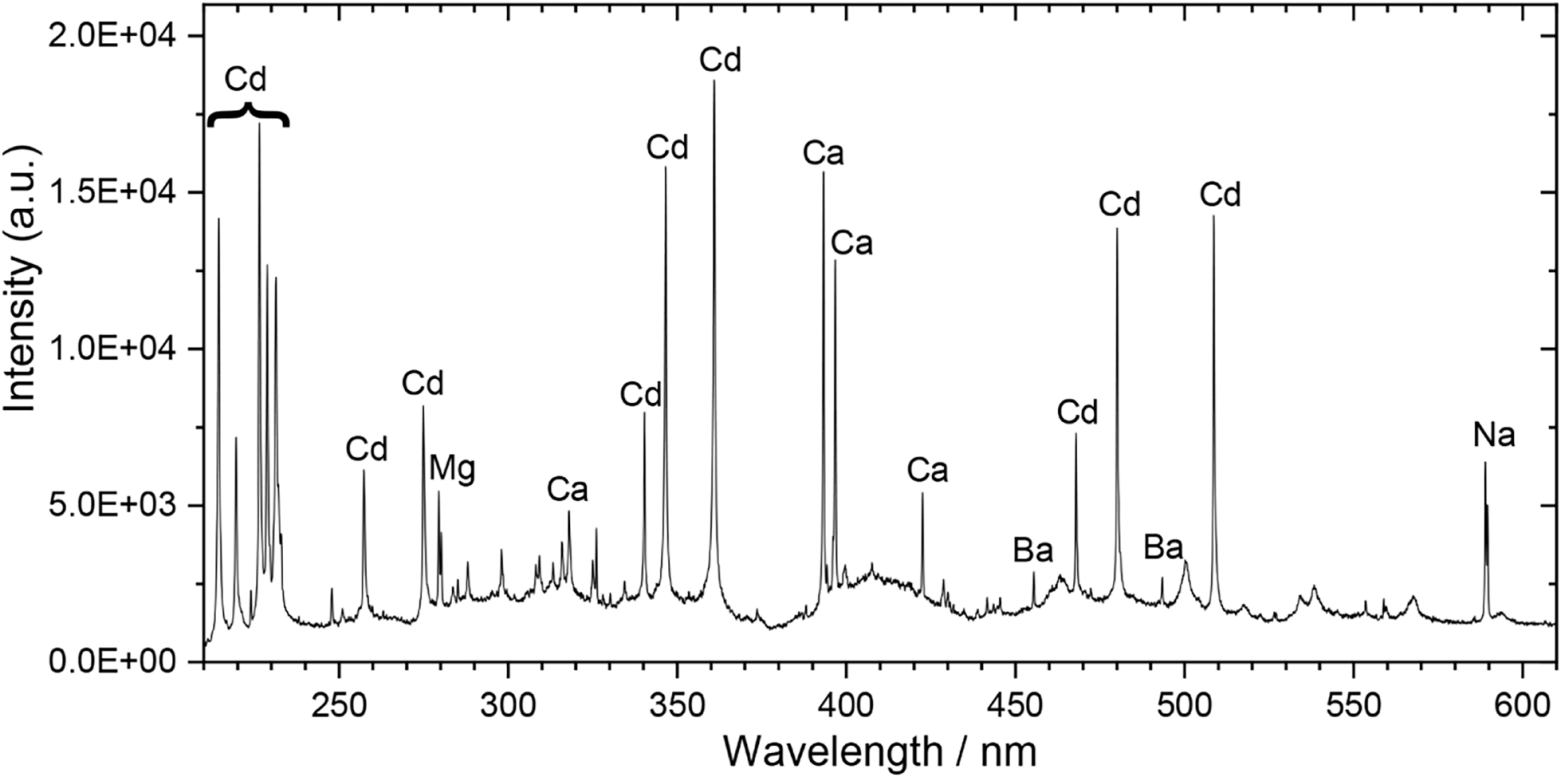
Single-shot LIBS spectrum of red paint (τ_DELAY_ = 1 μs, τ_G_ = 30 μs). The majority of the lines in the spectrum are attributed to cadmium, leading to the identification of the pigment as cadmium red.

In all, the examples shown highlight the capabilities and performance of the LIBS module integrated in the hybrid instrument and the type of multielemental information that can be obtained with a single-pulse analysis.

## Conclusions

This paper provides a detailed description of TriENA, a new hybrid portable instrument specifically designed and developed to meet common needs and requirements of Heritage Science research and practice works, with regard to materials analysis. The system integrates three complementary spectrochemical analysis techniques: Diffuse Reflectance, LED-Induced Fluorescence, and Laser-Induced Breakdown Spectroscopy. The instrument is compact consisting of two main components: a) an optical head (∼ 7 kg), housing the light sources, optics, and a viewing camera, which can be mounted on a tripod for convenient on-site analysis of objects and monuments and b) a control unit (∼10 kg), containing three compact spectrometers, power supplies, and the necessary electronics to drive the light sources in the unit head. The whole material can be easily packed and transported. Furthermore, the instrument design foresees several user-friendly features, such as an aiming laser diode and a viewing camera, which considerably simplify the procedure of positioning the object under investigation at the proper distance with respect to the optical head and selecting accurately the area to be analyzed. A plug-and-play accessory bracket facilitates quick positioning and removal of the Spectralon used to properly reference the diffuse reflectance measurements.

The integration of the three spectroscopy techniques allows the user to address various analytical questions and perform analyses of different types of objects and materials. The instrument’s performance was evaluated through several tests. Diffuse reflectance spectra of pigments on different substrates were acquired and compared to those obtained by using a benchtop commercial spectrometer. The similarity of the spectra validates the DR setup integrated into the hybrid device, confirming its efficacy and reliability. A complementary analysis of a fresco mock-up containing a diluted Egyptian blue pigment using both DR and LED-IF demonstrates the advantage of combining multiple techniques for the characterization of complex objects. In addition, LED-IF was employed for the detection of chlorophyll, and the results compare favorably against our previous work. The new system is more efficient and enables shorter integration times, reducing the influence of ambient light—a crucial advantage for field measurements where ambient light cannot be controlled. In the case of LIBS, the combined use of DR and LIBS enabled the unambiguous identification of a cadmium red pigment, illustrating how complementary analysis can overcome specific detection limitations of individual techniques, for example, the fact that S and Se emissions fall outside the spectral range of the LIBS spectrometer. In addition, the LIBS analysis of a bronze alloy showed that the system has a sensitive response across the entire spectral range (207–680 nm) and major and minor elements of the alloy were detected in a single-shot spectrum. The flexibility of the TriENA LIBS module permits the integration of additional spectrometers, potentially extending its spectral range to detect, for example, environmentally relevant elements such as nitrogen, sulfur, or chlorine, whose strongest emission lines fall outside the current detection window.

This compact and portable analytical tool is expected to have a significant role in the study and preservation of movable and immovable monuments at the Ephorate of Antiquities of the City of Athens, where it is currently located. Its routine use in the context of materials analysis, not only in the EACA laboratory but also at monument sites in the Athens area as well as at excavation sites, will strengthen the scientific support of important studies and conservation works. TriENA can also serve as a training tool for young scientists, conservators, and conservation professionals. Finally, following interaction with the EACA conservators, studies have been initiated on various archaeological and historical objects. In parallel, software routines, including machine learning tools, are being developed to expedite data processing and exploit as effectively as possible the spectrochemical data employed.

## Supplementary Material



## Notes

Table S1: list of emission lines labeled in the LIBS spectra shown in Figures 9 and 10 (PDF)
