# Supplementary material for: TriENA: a Portable, Hybrid Multimode Spectrometer Combining Diffuse Reflectance, LED-Induced Fluorescence, and Laser-Induced Breakdown Spectroscopy for a Holistic Analysis of Materials on Monuments and Objects of Archaeological Interest
Source: Anal Chem. 2025 Dec 5;97(49):27276–88. doi: 10.1021/acs.analchem.5c05236 (PMC12713606; doi:10.1021/acs.analchem.5c05236)
Supplement: Supplementary file 1 [file ac5c05236_si_001.pdf]

SUPPORTING INFORMATION

TriENA, a portable, hybrid multimode spectrometer combining Diffuse Reflectance, LED-Induced Fluorescence and Laser-Induced Breakdown Spectroscopy for a holistic analysis of materials on monuments and objects of archaeological interest

Victor Piñon [a*], Anastasia Giakoumaki [a], Michalis Andrianakis [a], Kostas Hatzigiannakis [a], Kristalia Melessanaki [a], Panagiotis Siozos [a], Demetrios Anglos [a, b*], Paraskevi Pouli [a]

[a] Institute of Electronic Structure and Laser, Foundation for Research and Technology - Hellas, Nikolaou Plastira 100, GR-700 13, Vassilika Vouton, Heraklion, Crete, Greece.

[b] Department of Chemistry, University of Crete, GR-710 03, Heraklion, Crete, Greece

* Email: vpinon@iesl.forth.gr

* Email: anglos@uoc.gr ; anglos@iesl.forth.gr

Table of Contents

Table S1: List of emission lines labelled in the LIBS spectra shown in Figures 9 and 10……………………………..…S2

Table S1: List of emission lines labelled in the LIBS spectra shown in Figures 9 and 10.

Roman numerals I and II next to element symbols indicate emissions from neutral and singly-ionized atoms respectively.

Bronze alloy (Figure 9)						Cadmium Red (Figure 10)			
Element	λ(nm)	Element	λ(nm)	Element	λ(nm)	Element	λ(nm)	Element	λ(nm)
Cu I	223.008	Cu II	210.480	Sn I	283.998	Cd I	228.802	Ca I	422.673
	224.427		213.598		285.062		340.365	Ca II	315.887
	324.754		219.227		286.331		346.620		317.933
	327.396	Pb I	280.199	Zn I	468.014		361.051		318.128
	465.112		282.319		472.216		467.815		393.366
	467.472		283.305		481.053		479.991		396.847
	470.459		287.331	Ca II	393.366		508.582	Mg II	279.553
	479.400		357.273		396.847	Cd II	214.439		280.270
	510.554		363.957	Na I	588.995		219.456	Ba II	455.403
	515.323		368.346		589.592		226.501		493.408
	521.820		373.993	Mg I	285.213		231.277	Na I	588.995
	570.024		405.781	Mg II	279.553		257.294		589.592
	578.213	Pb II	220.353		280.270		274.856		

Emission lines obtained from:

Kramida, A., Ralchenko, Yu., Reader, J. and NIST ASD Team (2024). NIST Atomic Spectra Database (version 5.12), [Online]. Available: https://physics.nist.gov/asd [Mon Aug 18 2025]. National Institute of Standards and Technology, Gaithersburg, MD. DOI: https://doi.org/10.18434/T4W30F
